# Cooking at Home, Fast Food, Meat Consumption, and Dietary Carbon Footprint among US Adults

**DOI:** 10.3390/ijerph19020853

**Published:** 2022-01-13

**Authors:** Julia A. Wolfson, Amelia M. Willits-Smith, Cindy W. Leung, Martin C. Heller, Donald Rose

**Affiliations:** 1 Department of International Health, Johns Hopkins Bloomberg School of Public Health, Baltimore, MD 21205, USA; 2 Department of Health Policy and Management, Johns Hopkins Bloomberg School of Public Health, Baltimore, MD 21205, USA; 3 Department of Social, Behavioral, and Population Sciences, Tulane University, New Orleans, LA 70112, USA; awillits@tulane.edu (A.M.W.-S.); diego@tulane.edu (D.R.); 4 Department of Nutritional Sciences, University of Michigan School of Public Health, Ann Arbor, MI 48109, USA; cinleung@umich.edu; 5 Center for Sustainable Systems, School for Environment and Sustainability, University of Michigan, Ann Arbor, MI 48109, USA; mcheller@umich.edu

**Keywords:** cooking, greenhouse gas emissions, sustainable diets, climate change, NHANES, dietary intake, dinner, meat

## Abstract

Shifting consumer behavior towards more sustainable diets can benefit environmental sustainability and human health. Although more frequent home cooking is associated with a better diet quality and fast-food consumption with worse diet quality, the environmental impact of diets based on frequency of cooking or eating fast food is not well understood. The objective of this study was to investigate whether the frequency of cooking dinner at home or eating fast food is associated with dietary greenhouse gas emissions (GHGE). We linked 24-h dietary recall data from adult respondents in the 2007–2010 National Health and Nutrition Examination Survey (NHANES) (N = 11,469) to a database of GHGE factors to obtain a measure of dietary GHGE (kgCO_2_-eq/2000 kcal) (the sum of emissions released in the production of food for an individual’s diet), adjusted by energy intake (kgCO_2_-eq/2000 kcal). We examined associations between frequency of cooking dinner (the only meal for which cooking frequency was measured), frequency of eating fast food, and dietary GHGE and protein sources (beef, pork, poultry, other meat, and fish and seafood (g/2000 kcal)) using generalized linearized regression models that controlled for age, sex, and other socio-economic characteristics. Greater cooking frequency was associated with higher dietary GHGE. In fully adjusted models, cooking 5–6 times/week was associated with an additional 0.058 kgCO_2_-eq/2000 kcal (SE 0.033) and cooking 7 times/week was associated with an additional 0.057 kgCO_2_-eq/2000 kcal (SE 0.027) when compared to cooking 0–2 times/week. Individuals in households who cooked dinner more frequently consumed significantly more meat, poultry, and fish (cooking 7 times/week: 148.7 g/2000 kcal vs. cooking 0–2 times/week: 135.4 g/2000 kcal, *p*-trend = 0.005), which could explain the association with a higher carbon footprint diet. There were few associations of note between fast-food frequency and GHGE. Policies and interventions that reduce consumption of meat and increase consumption of plants when both cooking meals at home and eating meals out are needed to shift toward diets that will be beneficial for both human health and the health of the planet.

## 1. Introduction

Climate change is one of the most pressing public health issues facing the world today. The modern food system and the agricultural sector are associated with numerous adverse environmental impacts and contribute an estimated 30% of global anthropogenic Greenhouse Gas Emissions (GHGE) [1,2,3]. Food animal production alone accounts for 14.5% of global GHGE [4], and the production of beef, for example, emits about 20 times the GHGE as some nuts, seeds, or legumes on an equivalent weight basis [5,6]. The effects of climate change will also have implications for food systems, nutrition, and food security across the globe, with, among other adverse outcomes, negative implications for crop yields, livestock productivity, reduced food security, and reduced nutrient density in many crops [7]. Food systems both contribute to climate change and will be seriously negatively impacted by climate change.

In the United States (US), meat consumption, and red meat consumption in particular, is consistently far above recommended levels based on national Dietary Guidelines [1,8,9]. Therefore, reducing meat consumption is increasingly promoted as a logical strategy to reduce food systems contributions to climate change. Evidence suggests that shifting current dietary patterns towards more sustainable diets, with reduced amounts of meat consumed, could reduce diet-related GHGE up to 55% [10].

Changing individual behavior towards more sustainable diets may also result in improvements to diet quality which remains poor in the US. Currently, children and adults consume too much meat, sugar, salt, and saturated fats, and too few fruits, vegetables, and whole grains [1]. A ‘planetary health’ diet lower in red meat and saturated fats and higher in fruits, vegetables, legumes, and whole grains [1], given current US dietary patterns, may necessitate meaningful changes to at-home meals as well as food choices when eating out.

Prior research has shown that the healthfulness of one’s diet is associated with differences in dietary GHGE; low-GHGE diets have higher Healthy Eating Index (HEI) scores than high-GHGE diets [11]. A growing body of evidence shows that cooking more frequently at home is associated with better diet quality, including higher HEI scores, and lower energy intake [12,13,14,15]. Conversely, a large body of evidence links away from home food sources, e.g., fast food and other restaurants, with lower diet quality, higher energy intake, bigger portion sizes, and higher obesity rates [16,17,18,19,20,21,22,23].

Public health efforts to improve diet quality have recently encouraged cooking at home as a means for healthier eating [8]. However, unless cooking more at home is also associated with lower meat consumption, particularly lower red meat consumption, encouraging more home cooked meals may not also result in lower GHGE diets. Prior evidence examining the association between cooking frequency and HEI-2015 shows that among low-income households cooking dinner more frequently is associated with higher total protein intake, and among high-income households, while cooking dinner more frequently at home is not associated with differences in total protein intake it is associated with greater intake of saturated fats [12]. Furthermore, eating at fast-food restaurants may also be associated with higher diet-related GHGE given large portion sizes [24], and that meat-based dishes comprise approximately four-fifths of items on fast food menus [25].

In this paper we build on prior work from Heller et al. [5] and Rose et al. [11] showing that better-quality diets are associated with lower GHGE, and work by Wolfson et al. [12] showing that more frequent cooking at home is also associated with better diet quality to investigate whether frequency of cooking dinner meals at home or frequency of eating at fast-food restaurants is associated with differences in diet-related GHGE. We focus on fast food in particular, rather than food away from home generally, due to the strength of the evidence linking fast food to poor diet quality [18]. We also make use of previous research linking environmental impact data to the dietary intake data in the National Health and Nutrition Examination Survey (NHANES) [26], a large, nationally representative survey of the US population [5,11]. Due to the previously documented evidence showing a positive relationship between cooking frequency and higher diet quality, and between lower-GHGE diets and better diet quality, we hypothesized that more frequent cooking at home would be associated with lower GHGE diets and more frequent eating at fast-food restaurants would be associated with higher GHGE diets.

## 2. Materials and Methods

This study is based on data obtained from the 2007–2010 NHANES, the only years in which NHANES included a question about frequency of cooking dinner [26]. The NHANES is a national, cross-sectional survey that uses a multistage, probability-based sampling strategy to achieve a nationally representative sample of the US civilian, noninstitutionalized population. NHANES participants answer questions about health-related behaviors and complete two 24 h dietary recalls. We used data from the first 24 h dietary recall, which is completed during the in-person interview with a trained NHANES health interviewer, who collects details about all foods and beverages consumed by respondents over the prior 24 h (from midnight to midnight). In the 2007–2008 and 2009–2010 survey cycles NHANES included a question about the weekly frequency of cooking dinner. More details about data collection procedures and analytic guidelines are available elsewhere [26]. Institutional Review Board approval was not needed as this was a secondary data analysis of publicly available, de-identified data.

### 2.1. Study Sample

The study sample included adults aged 18 years or older with complete and reliable data (as determined by NHANES staff) for the first in-person dietary recall. We excluded participants if they were pregnant at the time of data collection (*n* = 123) or if they were missing data for the cooking frequency measure (*n* = 138) or if they indicated they cooked dinner >7 times/week (*n* = 11). The final analytic sample included 11,469 adults.

### 2.2. Environmental Impacts and Linkage to Dietary Data

We assessed the GHGE released in the primary production of all foods and beverages reported by NHANES participants. The primary outcome measure was the overall daily GHGE/2000 kcal. Briefly, a database of environmental impacts of different foods was developed after extensive review of the life cycle assessment literature. Mean values of GHGE were calculated in kilograms of carbon dioxide equivalents (kg CO_2_eq) per kg of commodity (based on relevant studies of the literature on emissions from production, and in some cases, processing of foods) [5]. GHGE of foods were linked to NHANES dietary intake data using food codes in the Food Commodity Intake Database (FCID) [27]. Individual food items as eaten and reported in NHANES dietary recalls are translated into commodity form through thousands of recipes. For each recipe, we summed the GHGE of all the ingredients, adjusting for recipe quantities and amounts eaten to derive the GHGE for each food or dish reported by the NHANES participant. In some cases, GHGE were linked directly to NHANES foods. GHGE were then aggregated to an overall GHGE impact for the individual’s diet on the interview day. Additional details regarding how the GHGE of foods were calculated has been published previously [5,11]. Because of different energy needs of individuals and to focus our work on diet composition effects, GHGE for each individual were adjusted to a 2000 kcal diet. We also divided the overall measure of GHGE/2000 kcal into quintiles and created a dichotomous measure of being in the highest quintile of daily GHGE/2000 kcal compared to being in the lower four quintiles.

### 2.3. Meat, Poultry, and Fish Consumption

In addition to the primary outcome of daily GHGE/2000 kcal from dietary intake overall, we also examined the quantity (in grams/2000 kcal) of consumption of foods that are major contributors of GHGE [5]. We examined the quantity of meat, poultry, and fish and seafood consumption overall, as well as by specific sub-group items, including beef, pork, poultry, other meat (sheep, goat, rabbit, and game), and fish and seafood. These foods were identified from the FCID recipe files, which allows translation of NHANES as-consumed foods to commodities. Combined, these animal protein foods account for >60% of all diet-related GHGE [5].

### 2.4. Cooking Frequency

Household cooking frequency in NHANES was measured using the following survey question, “During the past seven days, how many times did you or someone else in your family cook food for dinner or supper at home?” The NHANES survey did not ask about frequency of cooking other meals. Following prior literature [12], cooking frequency was categorized into the following four groups: 0–2 times/week, 3–4 times/week, 5–6 times/week, >7 times/week.

### 2.5. Fast Food Frequency

Fast food consumption during the past 7 days was based on two NHANES questions. The first asked “During the past seven days, how many meals did you get that were prepared away from home in places such as restaurants, fast-food places, food stands, grocery stores, or from vending machines?” The second asked “How many of those meals did you get from a fast-food or pizza place?” Based on the distribution of the data, fast food frequency was categorized into the following four groups: 0 times/week, 1 time/week, 2–4 times/week, ≥5 times/week.

### 2.6. Additional Food Related Measures

Additional food-related measures related to cooking behavior and food choices were included as additional control variables. Specifically, we included consumption of ready-to-eat meals in the past 30 days (0 times/month, 1–4 times/month, ≥5 times/month), consumption of frozen meals/pizzas in the past 30 days (0 times/month, 1–7 times/month, ≥8 times/month), and the relative contribution of food at home vs. food away from home. To create this variable, we first divided calories consumed at home by total calories consumed then created a four-category variable (0–46.2%, 46.3–79.7%, 79.8–99.9%, 100%). All categorical variables of these food related measures were based on the distribution of the data.

### 2.7. Demographic, Socioeconomic, and Other Covariates

Covariates included sex (male, female), age (18–29 years, 30–49 years, 50–65 years, >65 years), race/ethnicity (Hispanic, non-Hispanic white, non-Hispanic Black, other/multiracial), education (<high school, high school graduate or equivalent, some college, college graduate or higher), household income (<100% federal poverty level (FPL; the FPL is the level of annual income for various household sizes set by the federal government, at which a household would be considered to be in poverty), 100 –< 200% FPL, 200 –< 500%FPL, ≥500% FPL), household size (1–3 people, ≥4 people), and employment status (employed, not working).

### 2.8. Statistical Analysis

All analyses used day 1 dietary sample weights as well as strata and primary sampling units (PSU) provided by NHANES staff to account for the unequal probability of being selected due to the complex sampling strategy, non-response for initial participation, and non-response for the dietary recall, and whether or not the 24 h dietary recall was for a weekend or weekday in order to produce national, representative estimates. First, we examined the distribution of sample characteristics by frequency of cooking dinner to assess bivariate associations between cooking frequency and demographic characteristics, dietary GHGE, and consumption of protein sources, and fast-food frequency and other food behaviors. We next used generalized linearized models (GLM) with a gamma family and log link to assess the association between cooking frequency and daily GHGE/2000 kcal and, separately, fast-food frequency and daily GHGE/200 kcal. This model specification was used due to the skewed nature of the outcome measures. We used four models: (1) unadjusted, (2) age- and sex-adjusted, (3) multivariable-adjusted, which included the demographic and socioeconomic covariates described above, and (4) model three covariates and the additional food covariates described above. Models 1–3 were estimated separately for cooking frequency and fast food frequency. Model 4 mutually adjusted for cooking and fast food frequency. We used post-estimation margins commands to estimate the mean predicted GHGE by cooking frequency and fast food frequency. Next, we used the third GLM model (separate models for fast food and cooking frequency both adjusted for socio-demographic measures) to estimate quantity of meat and fish consumption by cooking frequency and fast-food frequency. Finally, we used logistic regression models to estimate the odds of being in the highest quintile of daily GHGE/2000 kcal compared to the lower four quintiles by cooking frequency using the four models (unadjusted, age and sex adjusted, adjusted for socio-demographic characteristics, and mutually adjusted for cooking and fast food frequency and other food related measures) described above. Analyses were performed using Stata version 15.0 (StataCorp LP, College Station, TX, USA), all tests were two-sided and significance was considered at *p* < 0.05.

## 3. Results

Table 1 presents the characteristics of the study sample overall and stratified by cooking frequency. Overall, 13% of American adults ≥18 years old lived in households where dinner was cooked 0–2 times/week, whereas 36% lived in households where dinner was cooked 7 times/week. Household cooking frequency differed by sex, age, race/ethnicity, education, income, household size, and employment status (all *p* < 0.05). Less than 5% of the sample (*n* = 551) were missing data for fast-food frequency, evenly distributed across cooking frequency categories. Overall mean daily GHGE were 2.20 kgCO_2_/2000 kcal (SD 0.02). GHGE differed based on household cooking frequency (*p*-trend = 0.015). For individuals living in households where dinner was cooked the least often (0–2 times/week), mean GHGE was 2.10 kgCO_2_/2000 kcal (SD 0.05), whereas mean GHGE was 2.23 kgCO_2_/2000 kcal (SD 0.04) among households in which dinner was cooked the most frequently (7 times/week).

Results showing the associations between cooking frequency and fast-food frequency with GHGE are shown in Table 2. Higher cooking frequency was associated with greater GHGE in unadjusted models. Compared to cooking dinner 0–2 times/week, cooking dinner 5–6 times/week was associated with 0.058 kgCO_2_/2000 kcal more GHGE (SE 0.022) and cooking 7 times/week was associated with 0.061 kgCO_2_/2000 kcal (SE 0.025) more GHGE. This association persisted after adjustment for age and sex, and in multivariable adjusted models. In fully adjusted models, compared to cooking 0–2 times/week, cooking 5–6 times/week was associated with 0.058 kgCO_2_/2000 kcal (SE 0.033) more GHGE and cooking 7 times/week was associated with 0.057 kgCO_2_/2000 kcal (SE 0.027) more GHGE. There were no significant associations between frequency of consuming fast food and GHGE across all four models. In Model 4, for both cooking frequency and fast food frequency, results were robust to further adjustment for the proportion of total calories coming from food at home, frequency of, ready to eat meals, and frozen meals/pizza consumption. Full model results for Model 3 are available in Appendix A and Appendix B.

**Figure 1:**
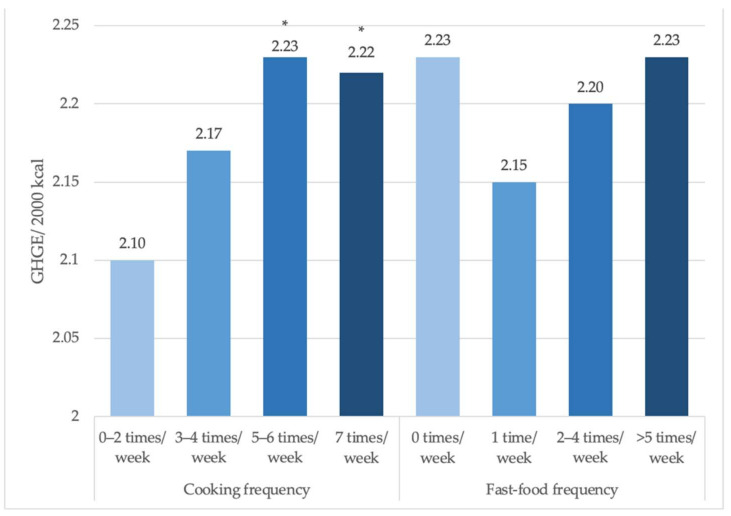
Predicted daily GHGE per 2000 kcal based on frequency of cooking dinner at home and eating out at fast food restaurants (NHANES 2007–2010). Note: Based on post-estimation margins following separate GLM models with gamma family and log link that controlled for age, sex, race/ethnicity, income, education, employment status, and household size. * *p* < 0.05. *p*-trend for cooking frequency = 0.03; *p*-trend for fast-food consumption = 0.719.

Figure 1 shows the predicted mean GHGE after models adjusted for socio-demographic characteristics (Model 3) for both cooking and fast-food frequency. Cooking dinner 0–2 times/week was associated with 2.10 kgCO_2_/2000 kcal which was lower than those cooking dinner 5–6 times/week (2.23 kgCO_2_/2000 kcal, *p* = 0.017) and those cooking dinner 7 times/week (2.22 kgCO_2_/2000 kcal, *p* = 0.041). There were no significant differences in mean GHGE based on frequency of eating fast food.

**Figure 2:**
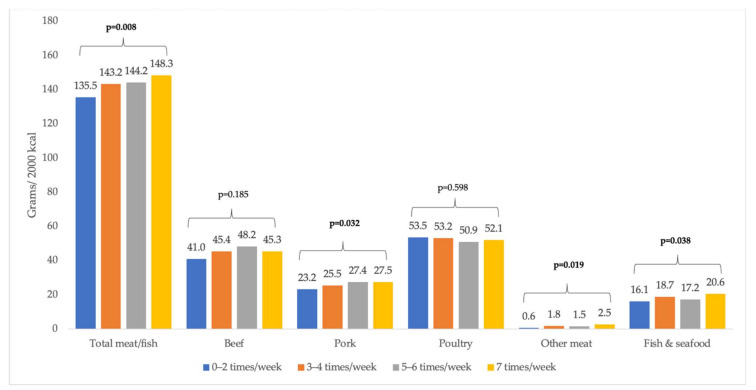
Meat and fish consumption (grams per 2000 kcal) by cooking frequency, NHANES 2007–2010. Note: Based on post estimation margins after GLM models with gamma family and log link adjusted for age, sex, race/ethnicity, income, education, employment status, and household size. Other meat includes: sheep, goat, rabbit, and game. Six separate models were run for this figure, one for each type of meat and one overall model for the total consumption amounts. *p*-value is for the linear trend.

Figure 2 shows the multivariable-adjusted mean consumption of meat (including beef, pork, other meat (sheep, goat, rabbit, and game)), poultry, and fish by cooking frequency. Overall, individuals in households who cooked dinner more frequently consumed significantly more meat, poultry, and fish (cooking 7 times/week: 148.3 g/2000 kcal vs. cooking 0–2 times/week: 135.5 g/2000 kcal, *p*-trend = 0.008). This overall difference was driven by differences in meats other than poultry, which showed no differences by cooking frequency. For example, after adjusting for all demographic and socio-economic variables, those who cooked dinner the most frequently (7 times/week) consumed more pork than those who cooked the least frequently (0–2 times/week), by 27.5 to 23.2 g/2000 kcal, respectively (*p*-trend = 0.032). Beef was consumed in the greatest quantities, and although there was not an overall trend effect throughout the range of cooking frequency (beef *p*-trend = 0.185), two-way comparisons indicate clear differences. For example, those who cooked dinner 5–6 times/week consumed more beef (48.2 g/2000 kcal) than those who cooked 0–2 times/week (41.0 grams/2000 kcal, *p* = 0.006 in the fully adjusted model) (Appendix C).

**Figure 3:**
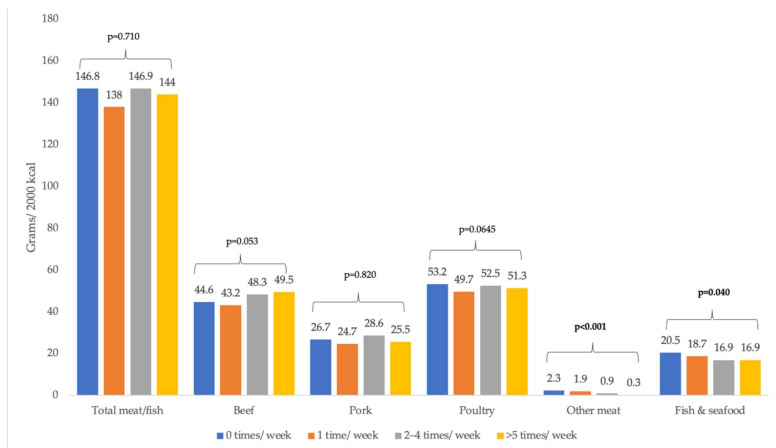
Meat and fish consumption (grams per 2000 kcal) by fast-food frequency, NHANES 2007–2010. Note: Based on post estimation margins after GLM models with gamma family and log link adjusted for age, sex, race/ethnicity, income, education, employment status, and household size. Other meat includes: sheep, goat, rabbit, and game. Six separate models were run for this figure, one for each type of meat and one overall model for the total consumption amounts. *p*-value is for the linear trend.

Figure 3 reports the multivariable-adjusted mean consumption of meat and fish by fast-food frequency. Differences in total meat and fish, pork, poultry were not significant. Those who ate fast food more frequently consumed more beef (*p*-trend = 0.053), and less other meat (*p*-trend < 0.001) and less fish and seafood (*p*-trend = 0.040) compared to those who ate fast food less frequently. Model results underlying the figure are available in Appendix D.

The odds of being in the highest quintile of GHGE/2000 kcal compared to the lower four quintiles by cooking frequency and fast-food frequency is presented in Table 3. In fully adjusted models, those who cook more frequently have significantly higher odds of being in the highest quintile of daily overall GHGE (cooking 5–6 times/week: OR 1.26 [95% CI: 1.01, 1.57]; cooking 7 times/week: OR 1.28 [95% CI: 1.03, 1.59]) compared to those who cook dinner 0–2 times/week. The magnitudes of these associations were consistent across models. Cooking 3–4 times/week was not significantly associated with differential odds of being in the highest quintile of GHGE. Across all models, frequency of eating fast food was not associated with odds of being in the highest quintile of GHGE.

## 4. Discussion

To our knowledge this is the first study to examine the relationship between frequency of cooking at home and frequency of eating fast food and diet-related environmental impacts in the US. We found that cooking at home more frequently was associated with consumption of a higher carbon footprint diet (GHGE/2000 kcal), which was the opposite of the relationship we had hypothesized *a priori*. The reason for the higher emission diet was largely driven by consumption of more beef, pork, and fish and seafood among people living in households where dinner was cooked more frequently. This result held true even when we controlled for the frequency of fast food, the proportion of total calories consumed at home, ready-to-eat foods, and frozen food consumption (Model 4). Also contrary to our hypothesis we did not find significant associations between fast-food consumption and GHGE. Beef and other ruminant animals are the largest contributors to food systems-related GHGE [1,4]. These results underscore how important it is to reduce meat consumption, even when cooking meals at home, as part of a strategy to lower food systems-related contributions to climate change. Changing behavior in the direction of less red meat consumption will have benefits for both climate change (via reduced GHGE) and for population health (via better aligning diets with dietary guidelines).

The present study builds on prior work using NHANES data showing that cooking more frequently at home is associated with a healthier diet, and that healthier diets are associated with lower GHGE [11,12]. Here, we found that cooking more frequently was associated with a slightly higher dietary carbon footprint, though the magnitude of our results is lower than other behavioural correlates of dietary GHGE. For example, Rose and colleagues [11] found that respondents who “tried national dietary guidance” had a dietary GHGE 3.6% lower than the mean, whereas our most frequent cooking group has a dietary GHGE about 2.6% higher than the least frequent cooking group. Additional evidence from both the US and other countries has shown that shifting diets towards greater alignment with dietary guidelines or healthier eating patterns would improve food systems-related environmental sustainability [5,28].

Our results are also consistent with existing evidence showing that diets higher in meat are associated with higher GHGE [29]. In our results, meat consumption, particularly pork, other meat, fish and seafood, and beef, was a key correlate of the higher GHGE associated with more frequent cooking. While cooking at home more frequently has been shown to be associated with better HEI scores, those differences were driven by higher total fruit, whole fruits, vegetables, and whole grains and not differences in protein foods [12]. Among lower-income individuals, for whom there is not as strong a correlation between cooking frequency and higher HEI, cooking more frequently was associated with higher total protein and seafood and plant proteins [12]. In the present study, lower education and lower income were both associated with cooking more frequently, and may therefore partially explain the association between cooking frequency and higher GHGE if these groups are also more likely to consume meat more frequently as well, as other evidence suggests [29]. In another study using food purchase and acquisition data from the US, households with lower socioeconomic status were more likely to purchase the highest amount of red meat as a share of total food spending [29]. Taken together, these findings suggest that while cooking at home more frequently may, indeed, be a strategy for consumption of an overall healthier diet, particularly for higher-income individuals, cooking more at home does not necessarily translate into a lower environmental impact unless additional changes, particularly reducing meat consumption, are also made.

It is notable that our results for the associations with GHGE for both cooking frequency and frequency of eating fast food were robust to the different model specifications we estimated. In particular, in addition to adjustment for socio-demographic characteristics, further adjustment for the percentage of total calories consumed at home (compared to away from home), ready-to-eat meals, frozen meals/pizzas, and mutual adjustment for fast food and cooking frequency did not alter results. Frequency of fast food remained nonsignificant and frequency of cooking remained significantly associated with higher GHGE, though the magnitude of the associations between frequent cooking at home and frequent fast-food consumption with GHGE was similar. This speaks to the primacy of the role meat plays in Americans’ diets regardless of where meals are being consumed or whether they are being cooked at home or eaten out in restaurants.

In the US, meat consumption is consistently far above recommended levels despite public health campaigns to promote more plant-based diets. People in the US consume far more meat, especially red meat, than any other region in the world [1]. Processed meat has been associated with increased risk of all-cause mortality and cardiovascular disease mortality, and strong evidence links consumption of red meat with increased risk of mortality, stroke, colon cancer, and type-2 diabetes [30,31,32]. Raising livestock for consumption as meat is associated not only with high GHGE, but is also associated with numerous harmful environmental impacts including water, air, and soil contamination [1,4]. Therefore, reducing meat consumption, when eating out and when cooking at home, represents a “win-win” dietary change that will have positive effects for both the individual’s health and the health of the planet [1]. In 2015, the Scientific Advisory Committee for the Dietary Guidelines for Americans included evidence in their report regarding food system environmental impacts, and concluded that diets higher in plant-based foods and lower in animal-based foods were more sustainable than the current US diet [33]. However, the final 2015 Dietary Guidelines for Americans did not include any discussion of environmental sustainability or food systems contributions to climate change [8]. Though evidence regarding the detrimental environmental impacts of meat, and corresponding benefits of plant-based diets, has only grown in recent years, that evidence was deemed out of scope of the 2020 Dietary Guidelines for Americans which did not include any discussion of or recommendations about steps to reduce food system contributions to climate change [34,35]. This presents an important policy change opportunity for future iterations of the Dietary Guidelines for Americans and other government nutrition programs and recommendations. Strong and clearly articulated recommendations for eating patterns that account for both individual health and environmental health impacts are urgently needed [36].

In addition to incorporating sustainability and environmental impacts into the Dietary Guidelines for Americans, strong public health campaigns focusing on reducing meat consumption and interventions to address barriers for doing so are needed. Though individual estimates vary, surveys show that around one-quarter to one-third of Americans have reduced their meat consumption in recent years [37,38]. The primary reasons Americans cite for reducing meat consumption are health (50%) and cost (51%); whereas only 12% of American adults cite the environmental impact of meat consumption as part of their motivation for reducing meat in their diets [37]. This presents an opportunity to promote and emphasize the contribution of meat production to climate change, particularly as the issue of climate change has become a more salient and prominent part of public discourse. The Meatless Monday campaign is one example of a successful effort to reduce meat consumption one day a week in home and other settings, which has combined messages about individual health and environmental sustainability [39]. Americans who have not reduced their meat consumption cite multiple reasons including perceptions that a healthy diet includes meat (32%); a meal is incomplete without meat (18%); and that they do not know how to cook meatless meals (7%) [37]. These insights are an opportunity for cooking skills interventions and public health messages to promote alternatives to meat, healthy plant-based and meat alternative meal options.

The restaurant industry is also an important context in which to promote reducing meat consumption in favor of plant-based diets. On a typical day, more than one-third of Americans eat out in fast food restaurants [22]. In the U.S., the restaurant industry has documented increasing consumer demand for vegan, vegetarian, and plant-based items, and several major fast food chains have recently introduced meat-free/meat-alternative menu items. Restaurant industry reports have identified increasing sustainability as among the top restaurant menu trends in 2020 [40]. Climate change impact menu labeling systems for the restaurant industry have been recently introduced and have the potential to encourage more sustainable food choices in restaurant settings [41]. In the present study, we did not see an association between frequency of eating fast food and diet-related GHGE, however, analyses using more current data may show a different outcome given the growing number of more sustainable menu options now available. However, fast food menus still feature few healthy items, and though vegetarian items have, on average, lower calories than non-vegetarian menu items, they are also higher in sugar and carbohydrates [25], so the overall impact on both diet quality and climate change is worthy of future evaluation.

Cooking at home is a focus of public health efforts to improve diet quality in the US, particularly among lower-income groups who often lack easy access to healthy foods and face other structural barriers to cooking healthy meals [42]. In particular, affordability and accessibility of healthy foods such as fresh fruits and vegetables, as well as lack of time for shopping and cooking are barriers for many people that predate the COVID-19 pandemic and have continued during the pandemic [43,44,45]. Cooking at home has increased during the COVID-19 pandemic, with some evidence suggesting that such a shift to more cooking will be associated with positive changes to diet quality [46]. Meat substitutes and meat-free menu options have become increasingly popular in the restaurant sector, but grocery stores remain the primary purchase location for processed meat, red meat, poultry, and fish and shellfish among Americans [9]. This suggests that while there is growing demand for meatless options when dining out [40], meat still plays a central role in meals Americans cook at home. There is an opportunity for nutrition and cooking skills interventions that aim to increase confidence and skills related to cooking at home to focus more on plant-based cooking and more sustainable food choices (e.g., alternatives to red meat) even if sustainability is not the primary goal of the intervention. An opportunity also exists to consider financial policies (e.g., subsidies), or store-level interventions (e.g., product placements, cooking/ingredient demonstrations) to promote plant-based, or reduced meat, meal options to American home cooks.

### Strengths and Limitations

This study has several strengths including use of a large and nationally representative data with from the ‘gold standard’ diet and nutrition survey in the United States, linking to a previously developed, rigorous GHGE measure, and our ability to adjust for several related food behavior and cooking measures that add robustness to the interpretation of our results. However, these results should be considered in light of several limitations. First, cooking frequency is a household measure of cooking frequency over the past 7 days, and dietary intake is measured at the individual level using a single 24 h dietary recall. Therefore, the GHGE of individual diets may not reflect the overall intake pattern over the entire 7-day period in which cooking was measured and may not accurately reflect the overall relationship between cooking frequency and dietary GHGE. Second, the cooking frequency measure is based on a single question that was only asked in two NHANES waves. NHANES also did not include measures about frequency of cooking other meals. However, other national surveys show that dinner is the most frequently cooked meal in the US [47,48], so the focus on dinner may provide a good picture of household cooking habits (though this may have changed during the COVID-19 pandemic). Relatedly, it is possible that since these data were collected, the relationship between cooking frequency and GHGE has shifted given greater promotion of plant-based diets since 2010. As has been discussed elsewhere [13,49], the cooking measure is also open to a great deal of interpretation and could limit understanding of the relationship between certain types of cooking at home and GHGE. However, our analyses further adjusting for fast food, ready-to-eat foods, and frozen foods (Model 4) were highly consistent with our results and mitigates some of this concern. Third, our measure of environmental impact focused only on GHGE/2000 kcal and was based on only ‘cradle-to-farm gate’ impacts. The reasons for this measurement approach are described elsewhere [11], but the result is that dietary GHGE as measured for this study are likely to be underestimates. We have also not considered the GHGE inherent in the process of home cooking itself, which includes transportation to purchase foods (e.g., driving to grocery stores or restaurants which may be higher for individuals living in rural areas), food storage, food cooking, storage of leftovers and waste [50]. However, there is no reason to believe the underestimation of GHGE would differ systematically between cooking frequency categories and there are mixed findings as to whether institutional cooking (e.g., store- or restaurant-purchased meals) is more efficient than home-cooked meals [51]. Finally, the notable variability in GHGE estimates of different foods has not been accounted for in this analysis. Previous work demonstrated that variability across life-cycle assessment studies introduced a ±19% range on the mean of individual diet GHGE [5]. Other evidence shows that variability due to agricultural production practices and location is typically greater for animal-based foods than for vegetable substitutes [6].

## 5. Conclusions

Contrary to our hypotheses, in this study we found that cooking dinner frequently at home is associated with higher diet-related GHGE (kgCO_2_/2000 kcal), and frequency of eating fast food is not associated with GHGE. The higher GHGE among people in higher cooking frequency households is driven by higher consumption of meat, particularly beef, pork, and fish/seafood. Policies and interventions that promote plant-based diets and reducing meat consumption when cooking meals at home and when eating out are needed to shift toward “win-win” diets that will be beneficial for both human health and the health of the planet.

## Figures and Tables

**Table 1:** Characteristics of the study sample, by frequency of cooking dinner, NHANES 2007–2010.

		Frequency of Cooking Dinner				
	Overall	0–2 Times/Week	3–4 Time/Week	5–6 Times/Week	7 Times/Week	*p*-Value
	N = 11,469	*n* = 1552	N = 2149	*n* = 2858	*n* = 4910	
	%					
Total	100	13.1	21.4	29.6	35.9	
Sex						
Female	51.6	12.1	21.4	30.3	36.2	0.037
Male	48.4	14.1	21.4	29.0	35.5	
Age						
18–29 years	21.7	16.6	24.2	28.2	30.9	**<0.001**
30–49 years	36.5	10.6	23.7	31.1	34.6	
50–65 years	26.3	13.5	20.4	29.9	36.2	
66+ years	15.5	13.4	13.8	27.7	45.1	
Race/ethnicity						
Hispanic	13.4	12.8	12.5	18.3	56.3	**<0.001**
Non-Hispanic white	69.5	12.1	22.6	34.0	31.4	
Non-Hispanic Black	11.4	20.4	27.6	20.8	31.1	
Other, multiracial	5.7	11.4	15.7	21.0	52.0	
Education						
<High school	19.9	12.1	14.7	20.4	52.8	**<0.001**
High school grad or equivalent	24.5	13.2	22.9	27.4	36.6	
Some college	30.3	13.9	22.5	31.9	31.7	
College grad or higher	25.3	12.8	24.0	36.3	26.8	
Income						
<100% FPL	14.4	15.4	15.9	18.0	50.8	**<0.001**
100–<200% FPL	19.2	13.0	17.6	23.0	46.4	
200–<500% FPL	35.4	12.0	24.7	32.4	30.9	
≥500% FPL	24.2	13.8	24.7	38.9	22.6	
Missing	6.8	11.5	15.2	25.6	47.7	
Household size						
1–3 people	64.7	16.3	22.4	28.4	32.9	**<0.001**
≥4 people	35.3	7.2	19.6	31.9	41.3	
Employment status						
Employed	61.5	13.4	24.5	31.3	30.9	<0.001
Not working	38.5	12.6	16.5	27.1	43.9	
Fast-food consumption ^a^						
0 times/week	44.4	10.2	15.1	25.8	48.9	**<0.001**
1 time/week	21.1	10.4	21.0	37.2	31.4	
2–4 times/week	23.7	12.7	30.7	33.3	23.3	
≥5 times/week	10.8	29.5	27.2	24.4	18.8	
% Calories consumed at home ^b,c^						
0–46.2%	26.7	18.1	23.8	30.4	27.7	**<0.001**
46.3–79.7%	27.5	11.9	23.3	33.3	31.5	
79.8–99.9%	10.8	10.5	21.7	31.5	36.3	
100%	35.0	10.5	17.8	26.2	45.5	
Ready-to-eat meals ^d^						
0 times/month	66.7	12.4	18.8	28.2	40.6	**<0.001**
1–4 times/month	23.0	11.7	23.8	36.3	28.2	
≥5 times/month	10.3	19.0	32.2	26.1	22.7	
Frozen meals/pizza ^e^						
0 times/month	54.4	12.4	18.1	27.8	41.6	**<0.001**
1–7 times/month	33.7	11.2	24.0	35.0	29.9	
≥8 times/month	11.9	19.9	28.5	24.9	26.6	
	Mean (SD)					
Daily GHGE/2000 kcal	2.20 (0.02)	2.10 (0.05)	2.16 (0.05)	2.23 (0.03)	2.23 (0.04)	**0.015**
Total meat and fish consumed (g/2000 kcal)	144.4 (1.9)	138.0 (4.4)	144.2 (3.7)	141.8 (2.6)	148.9 (2.6)	**0.042**
Beef consumed (g/2000 kcal)	45.6 (1.2)	41.1 (2.0)	45.2 (2.2)	47.6 (1.3)	45.9 (2.1)	**0.094**
Pork consumed (g/2000 kcal)	26.5 (0.8)	23.7 (1.8)	25.0 (1.5)	26.3 (1.3)	28.5 (1.2)	**0.012**
Poultry consumed (g/2000 kcal)	52.2 (1.6)	56.0 (4.3)	54.6 (2.5)	49.8 (1.9)	51.3 (1.8)	**0.120**
Other meat consumed (g/2000 kcal)	1.53 (0.3)	0.8 (0.4)	1.4 (0.4)	1.3 (2.3)	2.07 (0.6)	**0.113**
Fish/seafood consumed (g/2000 kcal)	18.5 (0.8)	16.4 (1.7)	18.0 (2.3)	16.8 (1.4)	21.1 (1.2)	**0.022**
Dairy consumed (g/2000 kcal)	242.0 (4.6)	238.4 (9.6)	238.1 (7.9)	243.2 (10.2)	244.8 (6.8)	**0.526**

Note: *p*-values from categorical variables are based on chi-squared tests. Significant *p*-values (<0.05) are bolded. *p*-value for continuous daily GHGE/2000 kcal is the *p*-trend from simple ordinary least squares (OLS) regression. Column percentages shown for the overall column; row percentages shown for distribution of demographic measures by cooking frequency. ^a^ total N = 10,918. ^b^ Cut points based on the distribution of the data roughly equivalent to quartiles. ^c^ total N = 10,919; ^d^ total N = 10,902; ^e^ total N = 10,905; FPL= federal poverty level.

**Table 2:** Daily GHGE per 2000 kcal by cooking frequency and fast-food frequency, NHANES 2007–2010.

	Model 1: Unadjusted			Model 2: Age & Sex Adjusted			Model 3: Multivariable Adjusted for Socio-Demographics			Model 4: Multivariable Adjusted for Socio-Demographics and Food Behaviors		
	Coef. (SE)	*p*-Value	*p*-Trend	Coef. (SE)	*p*-Value	*p*-Trend	Coef. (SE)	*p*-Value	*p*-Trend	Coef. (SE)	*p*-Value	*p*-Trend
Cooking frequency												
0–2 times/week	[ref]			[ref]			[ref]			[ref]		
3–4 times/week	0.030 (0.033)	0.379	**0.015**	0.032 (0.033)	0.346	**0.021**	0.030 (0.033)	0.371	**0.030**	0.032 (0.035)	0.360	**0.039**
5–6 times/week	0.058 (0.022)	**0.014**		0.059 (0.022)	**0.012**		0.058 (0.023)	**0.018**		0.059 (0.027)	**0.038**	
7 times/week	0.061 (0.025)	**0.020**		0.060 (0.025)	**0.025**		0.057 (0.027)	**0.042**		0.055 (0.029)	0.064	
Fast-food consumption												
0 times/week	[ref]			[ref]			[ref]			[ref]		
1 time/week	−0.043 (0.023)	0.075	0.863	−0.039 (0.025)	0.128	0.789	−0.040 (0.026)	0.138	0.719	−0.036 (0.026)	0.172	0.761
2–4 times/week	−0.015 (0.020)	0.445		−0.014 (0.023)	0.559		−0.016 (0.024)	0.515		−0.006 (0.023)	0.792	
≥5 times/week	0.010 (0.026)	0.694		0.003	0.915		0.000 (0.029)	0.997		0.019 (0.029)	0.518	

Note: Separate GLM models with gamma family and log link (Models 1–3). Multivariable adjusted Model 3 controlled for age, sex, race/ethnicity, income, education, employment status, and household size. Model 4 controlled for Model 3 covariates and cooking frequency, fast-food frequency, percent of total calories consumed at home, ready-to-eat meals, and frozen meals/pizzas. [ref] refers to the reference group. Significant *p*-values (<0.05) are bolded.

**Table 3:** Odds of being in highest quintile of GHGE per 2000 kcal by cooking frequency and fast-food frequency, NHANES 2007–2010.

	Model 1: Unadjusted		Model 2: Age & Sex Adjusted		Model 3: Multivariable Adjusted for Socio-Demographics		Model 4: Multivariable Adjusted for Socio-Demographics and Food Behaviors	
	OR [95% CI]	*p*-Value	OR [95% CI]	*p*-Value	OR [95% CI]	*p*-Value	OR [95% CI]	*p*-Value
Cooking frequency								
0–2 times/week	[ref]		[ref]		[ref]		[ref]	
3–4 times/week	1.18 [0.90, 1.53]	0.218	1.19 [0.91, 1.56]	0.194	1.19 [0.90, 1.57]	0.207	1.20 [0.89, 1.63]	0.223
5–6 times/week	1.25 [1.02, 1.52]	**0.031**	1.26 [1.03, 1.53]	**0.026**	1.26 [1.01, 1.57]	**0.040**	1.27 [0.97, 1.65]	0.082
7 times/week	1.30 [1.07, 1.57]	**0.011**	1.30 [1.06, 1.58]	**0.013**	1.28 [1.03, 1.59]	**0.025**	1.28 [1.01, 1.61]	**0.040**
Fast-food consumption								
0 times/week	[ref]		[ref]		[ref]		[ref]	
1 time/week	0.83 [0.68, 1.02]	0.071	0.84 [0.69, 1.04]	0.103	0.84 [0.67, 1.04]	0.104	0.85 [0.69, 1.04]	0.116
2–4 times/week	0.91 [0.76, 1.10]	0.313	0.91 [0.74, 1.12]	0.379	0.91 [0.74, 1.13]	0.388	0.94 [0.77, 1.16]	0.567
≥5 times/week	1.01 [0.78, 1.30]	0.968	0.98 [0.75, 1.28]	0.861	0.96 [0.73, 1.27]	0.789	1.03 [0.78, 1.36]	0.834

Note: Separate logit models comparing highest quintile vs. lower four quartiles of emissions (Models 1–3). Multivariable adjusted Model 3 controlled for age, sex, race/ethnicity, income, education, employment status, and household size. Model 4 controlled for Model 3 covariates and cooking frequency, fast-food frequency, percent of total calories consumed at home, ready-to-eat meals, and frozen meals/pizzas. [ref] refers to the reference group. Significant *p*-values (<0.05) are bolded.

## Data Availability

NHANES data used for this study are publicly available at https://wwwn.cdc.gov/nchs/nhanes/ (accessed on 22 December 2021).

## Appendix A

**Table A1:** Full Model Results for Daily GHGE Per 2000 Kcal by Cooking Frequency, NHANES 2007–2010.

	Coef.	Std. Err.	t	*p* > t	95% Conf. Interval	
Cooking frequency						
0–2 times/week	[Ref]					
3–4 times/week	0.030	0.033	0.91	0.37	−0.038	0.098
5–6 times/week	0.058	0.023	2.51	**0.017**	0.011	0.106
7 times/week	0.057	0.027	2.13	**0.041**	0.002	0.111
Sex						
Female	[Ref]					
Male	0.084	0.016	5.21	**0.000**	0.051	0.117
Age						
18–29 years	[Ref]					
30–49 years	0.061	0.023	2.62	**0.013**	0.014	0.109
50–65 years	0.040	0.029	1.39	0.173	−0.019	0.099
66+ years	0.057	0.033	1.72	0.095	−0.010	0.125
Race/ethnicity						
Hispanic	[Ref]					
Non-Hispanic white	0.032	0.020	1.58	0.123	−0.009	0.073
Non-Hispanic Black	0.002	0.029	0.07	0.947	−0.057	0.061
Other, multiracial	0.008	0.051	0.15	0.883	−0.097	0.112
Education						
<High school	[Ref]					
High school grad or equivalent	0.000	0.027	0.01	0.993	−0.054	0.055
Some college	−0.041	0.028	−1.5	0.143	−0.097	0.015
College grad or higher	−0.045	0.022	−2.06	0.047	−0.090	−0.001
Income						
<100% FPL	[Ref]					
100–<200% FPL	−0.013	0.033	−0.41	0.685	−0.080	0.053
200–<500% FPL	−0.011	0.022	−0.48	0.638	−0.056	0.035
≥500% FPL	−0.021	0.029	−0.74	0.465	−0.080	0.038
Missing	−0.046	0.049	−0.95	0.348	−0.146	0.053
Household size						
1–3 people	[Ref]					
≥4 people	−0.009	0.019	−0.47	0.641	−0.049	0.030
Employment status						
Not working	[Ref]					
Employed	−0.010	0.024	−0.43	0.672	−0.059	0.039
Constant	0.684	0.041	16.57	0	0.600	0.768

Note: GLM models with gamma family and log link adjusted for age, sex, race/ethnicity, income, education, employment status, and household size. Significant *p*-values (<0.05) are bolded.

## Appendix B

**Table A2:** Full Model Results for Daily GHGE Per 2000 Kcal by Fast Food Frequency, NHANES 2007–2010.

	Coef.	Std. Err.	t	*p* > t	95% Conf. Interval	
Fast-food consumption						
0 times/week	[Ref]					
1 time/week	−0.040	0.026	−1.52	0.138	−0.094	0.014
2–4 times/week	−0.016	0.024	−0.66	0.515	−0.064	0.033
≥5 times/week	0.000	0.029	0	0.997	−0.060	0.060
Sex						
Female	[Ref]					
Male	0.080	0.015	5.39	**0.000**	0.050	0.110
Age						
18–29 years	[Ref]					
30–49 years	0.045	0.025	1.8	0.081	−0.006	0.096
50–65 years	0.024	0.034	0.71	0.481	−0.045	0.093
66+ years	0.036	0.039	0.94	0.355	−0.042	0.115
Race/ethnicity						
Hispanic	[Ref]					
Non-Hispanic white	0.040	0.022	1.84	0.075	−0.004	0.084
Non-Hispanic Black	0.001	0.031	0.03	0.974	−0.062	0.065
Other, multiracial	0.013	0.053	0.24	0.809	−0.095	0.121
Education						
<High school	[Ref]					
High school grad or equivalent	−0.008	0.027	−0.29	0.771	−0.062	0.046
Some college	−0.048	0.027	−1.79	0.084	−0.102	0.007
College grad or higher	−0.055	0.021	−2.66	**0.012**	−0.096	−0.013
Income						
<100% FPL	[Ref]					
100–<200% FPL	−0.006	0.034	−0.17	0.867	−0.074	0.063
200–<500% FPL	−0.002	0.022	−0.07	0.944	−0.046	0.043
≥500% FPL	−0.013	0.029	−0.45	0.658	−0.072	0.046
Missing	−0.028	0.048	−0.59	0.557	−0.125	0.069
Household size						
1–3 people	[Ref]					
≥4 people	0.004	0.019	0.22	0.831	−0.034	0.042
Employment status						
Not working	[Ref]					
Employed	−0.019	0.027	−0.7	0.487	−0.073	0.036
Constant	0.753	0.040	18.62	0	0.671	0.835

Note: GLM models with gamma family and log link adjusted for age, sex, race/ethnicity, income, education, employment status, and household size. [ref] refers to the reference group. Significant *p*-values (<0.05) are bolded.

## Appendix C

**Table A3:** Meat, Fish, and Dairy Consumption (Grams Per 2000 kcal) by Cooking Frequency, NHANES 2007–2010.

	Model 3: Multivariable Adjusted for Socio-Demographics		
	Predicted Grams (SE)	*p*-Value	*p*-Trend
Total meat/fish			
Cook 0–2 times/week	135.48 (4.25)	[ref]	**0.008**
Cook 3–4 times/week	143.21 (3.54)	0.159	
Cook 5–6 times/week	144.16 (2.61)	0.081	
Cook 7 times/week	148.34 (2.47)	**0.009**	
Beef			
Cook 0–2 times/week	41.01 (2.06)	[ref]	0.185
Cook 3–4 times/week	45.36 (1.97)	0.169	
Cook 5–6 times/week	48.20 (1.35)	**0.006**	
Cook 7 times/week	45.26 (2.26)	0.126	
Pork			
Cook 0–2 times/week	23.25 (1.86)	[ref]	**0.032**
Cook 3–4 times/week	25.55 (1.52)	0.388	
Cook 5–6 times/week	27.36 (1.42)	0.072	
Cook 7 times/week	27.55 (1.24)	0.055	
Poultry			
Cook 0–2 times/week	53.49 (4.05)	[ref]	0.598
Cook 3–4 times/week	53.22 (2.22)	0.953	
Cook 5–6 times/week	50.95 (1.89)	0.568	
Cook 7 times/week	52.15 (1.59)	0.739	
Other meat			
Cook 0–2 times/week	0.62 (0.24)	[ref]	**0.019**
Cook 3–4 times/week	1.83 (0.55)	**0.027**	
Cook 5–6 times/week	1.52 (0.28)	**0.030**	
Cook 7 times/week	2.51 (0.64)	**0.001**	
Fish and Seafood			
Cook 0–2 times/week	16.09 (1.66)	[ref]	**0.038**
Cook 3–4 times/week	18.66 (2.12)	0.376	
Cook 5–6 times/week	17.24 (1.62)	0.551	
Cook 7 times/week	20.59 (1.30)	**0.042**	

Note: GLM model with gamma family and log link. Model 3 controlled for age, sex, race/ethnicity, income, education, employment status, and household size. Other meat includes: sheep, goat, rabbit, and game. Model 3 is the data underlying Figure 1. [ref] refers to the reference group. Significant *p*-values (<0.05) are bolded.

## Appendix D

**Table A4:** Meat, Fish and Dairy Consumption (Grams Per 2000 kcal) by Fast Food Frequency, NHANES 2007–2010.

	Model 3: Multivariable Adjusted for Socio-Demographics		
	Predicted Grams (SE)	***p***-Value	*p*-Trend
Total meat/fish			
Fast-food 0 times/week	146.76 (2.14)	[ref]	0.710
Fast-food 1 time/week	138.04 (3.62)	**0.025**	
Fast-food 2–4 times/week	146.92 (2.48)	0.959	
Fast-food ≥5 times/week	144.01 (4.16)	0.546	
Beef			
Fast-food 0 times/week	44.59 (1.98)	[ref]	0.053
Fast-food 1 time/week	43.16 (1.82)	0.571	
Fast-food 2–4 times/week	48.27 (1.62)	0.124	
Fast-food ≥5 times/week	49.51 (2.87)	0.143	
Pork			
Fast-food 0 times/week	26.71 (1.48)	[ref]	0.820
Fast-food 1 time/week	24.74 (1.62)	0.306	
Fast-food 2–4 times/week	28.60 (1.03)	0.333	
Fast-food ≥5 times/week	25.53 (2.18)	0.680	
Poultry			
Fast-food 0 times/week	53.20 (1.99)	[ref]	0.645
Fast-food 1 time/week	49.73 (1.97)	0.073	
Fast-food 2–4 times/week	52.51 (2.30)	0.807	
Fast-food ≥5 times/week	51.34 (4.07)	0.672	
Other meat			
Fast-food 0 times/week	2.33 (0.47)	[ref]	**<0.001**
Fast-food 1 time/week	1.85 (0.57)	0.397	
Fast-food 2–4 times/week	0.91 (0.27)	**0.016**	
Fast-food ≥5 times/week	0.28 (0.09)	**<0.001**	
Fish and Seafood			
Fast-food 0 times/week	20.48 (1.04)	[ref]	**0.040**
Fast-food 1 time/week	18.66 (2.05)	0.433	
Fast-food 2–4 times/week	16.94 (1.46)	**0.046**	
Fast-food ≥5 times/week	16.91 (2.05)	0.146	

Note: GLM model with gamma family and log link. Multivariable adjusted Model 3 controlled for age, sex, race/ethnicity, income, education, employment status and household size. Other meat includes: sheep, goat, rabbit, and game. Model 3 is the data underlying Figure 2. [ref] refers to the reference group. Significant *p*-values (<0.05) are bolded.
